# Osteo-Porosis1Read before the Long Island Veterinary Society.

**Published:** 1890-12

**Authors:** George H. Berns


### OSTEO-POROSIS.[1]

BY DR. GEORGE H. BERNS.

AMONG the many diseases that a practising veterinarian meets with, I know of none that terminates more unsatisfactory to the owners of live stock, and is more annoying and of less credit to the practitioner than the complaint known as *osteo-porosis*, or big head.

Hundreds of animals are annually lost in this city from this disease, and the veterinarian, when called in, has, in the majority of instances, but little difficulty to diagnose the cases; but, unfortunately, that is all he can do. He can be of little service to the animal, and, from our present knowledge, cannot even give the owner satisfactory information as to its probable causes, nor advise preventive measures. An interchange of news and free discussion upon this subject by the members of this society will, no doubt, bring out many points of interest and value, and, with this object in view, I have prepared these few lines.

Upon symptoms, excepting a few premonitory ones, I will waste no time, for the characteristic enlargements of the bones of the face and lower jaw have been frequently seen and recognized by all; but in many instances we are called weeks or even months before any such well-developed symptoms exist. We find a horse lame on the near hind leg, with a history that he has been lame for some time, starts off on his toe when first leaving the stable, and warms out of it after he travels a while.

We carefully examine the hock, pastern and foot, but fail to find anything abnormal, excepting a slight coarseness of the hock. Under the circumstances, we consider ourselves justified to diagnose spavin, and fire and blister the hock and order the patient turned out to pasture for one or two months. In three or four weeks after the operation we are notified that our patient is not doing well, and are requested to visit him again in the country. We find him lame in the left shoulder this time; otherwise, apparently all right. We attribute the shoulder lameness to some accidental injury, or, perhaps, rheumatism; prescribe a mild lini-

[1]Read before the Long Island Veterinary Society.

ment, and advise the owner to keep his horse tied up in a dry stable for a short time.

Two or three weeks later we are called again, and find the patient all crippled up across the loins. We begin to look for enlargement of the lower jaw, but are unable to express a positive opinion; advise anti-rheumatic treatment. Horse taken up and placed in a good dry stable. Two or three weeks later we are called again, and find the horse with a peculiar elongated condition of the thigh, and the point of hock coming down to within six or eight inches of the ground—when forced to place weight upon the affected limb—and with well-marked enlargement of lower jaw. Of course, the case is plain enough. We have made a mistake in our original diagnosis, and caused the owner to spend considerable money upon an incurable patient.

The premonitory symptoms, therefore, are of the greatest importance; stiffness across the loins, causing the animal's gait to be of a peculiar rigid character is, in my opinion, the first and probably the most suggestive premonitory symptom in the majority of cases; next of importance is obscure lameness, particularly if more than one limb is involved. While I do not wish to say that lumbago and rheumatic lameness does not exist in horses, I am satisfied that 75 per cent. of all the cases diagnosed as such will turn out to be cases of osteo-porosis. In many cases a general unthrifty condition of the animal, and partial loss of appetite without any apparent cause, may also be noticed in the early stages. English veterinarians have evidently given the subject considerable attention; a large number or cases are on record, and the symptoms, post-mortem appearances, as well as the structural changes in the bones as revealed by the microscopic and chemical analysis, have been carefully studied and minutely described by Professor Varnell, Dr. Harly, Professor Williams and others. Germany, the acknowledged centre of all scientific investigations, has, as far as I have been able to ascertain, contributed little or nothing to the knowledge of this disease, and it is, therefore, reasonable to infer that it cannot exist in that country to any great extent. Now, let us speculate upon the probable causes, and consider the length of time it takes to develop. The English, basing their opinion largely upon animals out on pasture or confined in farm stables, seem to think that defective assimilation and malnutrition are the probable causes, and Prof. Williams in his surgery finishes his very able article on the subject by saying: "In all probability it will be discovered that the disease is due to the absence of some essential ingredient of the food, or its presence in such small quantities as to be insufficient for the necessities of the animal, or the presence of some other ingredient in such superabundance as to destroy the perfect composition of the whole, and thus render it unfit for assimilation, or so change it as to cause it to become even an irritant in the osseous system of the animal." As I have not been able to find the subject referred to in any other work at my command, I take it for granted that his views upon this disease are probably generally accepted by English veterinarians.

My experience with the disease, taking it from a practical point of view, compels me to question the soundness of Prof. Williams' opinion; I have seen, and, in some instances, observed for months, a very large number of cases in this city. Some years ago, when this disease was not as well understood and not as popular (if I may use the term) among veterinarians as it is now, it was my good fortune, or misfortune, I hardly know which, to come in contact with some ten or twelve cases inside of a few months.

In 1897, shortly after I graduated, I saw my first case of osteo-porosis, and there being one or two interesting events connected with it, I will here briefly refer to it. A bay gelding, 6 years old, the property of a well-known grocer, had been attended by a self-educated veterinarian of good standing, for a number of weeks for intermittent lameness, distemper and a peculiar swelling of the head, to which the owner had repeatedly called his attention, and for which a liniment had been prescribed. When I was called in to see him, some eight or ten days after the above referred to practitioner had discharged him as being cured, the animal presented all the symptoms of an ordinary acute case of influenza, but upon placing my finger upon the submaxillary artery I was surprised at the immense thickness of the inferior maxilla, and viewing the side of his face I could not help but notice a peculiar fullness under the eyes. In fact, the animal's head presented a very striking appearance even to an ordinary observer. Questioning the owner elicited the above history. A diagnosis of osteo-porosi was made, and the owner informed that his horse would very probably speedily recover from the influenza, but would advise him to dispose of him as soon as he was well, for the other complaint was positively incurable, and would rendert he animal useless for the remainder of his life. I based my opinion purely upon lectures heard at college and Prof. Williams' writings upon the subject. A few days later, meeting Dr. H. Hausman, as you all well know a regular graduate in veterinary medicine and an able practitioner of probably thirty or forty years' standing, having practised in this city to my knowledge for about twenty years, I related the case to him. He manifested considerable interest in the same and expressed a desire to see it. We visited the patient the next day, and the doctor admitted that it was a peculiar case, that he had never seen one before, and asserted that if he had been called in he would have certainly put the animal under experimental treatment, as the case was of such a nunusual character.

I willingly consented to place the patient under his care, and he experimented with him for all of four weeks ; at the expiration of that time, the horse being no longer able to masticate his food from irregular chisel-shaped molars which lacerated the palate, he was cast for operation upon his teeth, and being unable to rise again was either destroyed or died of exhaustion. The horse's head I prepared for a specimen, and it is still in my possession. Since that time I venture to say that over 200 cases have come under my observation in practice. I not only became deeply interested in the disease but quite enthusiastic, and at one time could see osteo-porosis in horses where no one else could, the result being that quite a few extremely embarrassing mistakes were made in diagnosis. Without exception, every one of the 200 or more cases were found in cellar stables, or in stables on the level of the street without cellars under them. Most all of them were old stables that had been used for years with the floors resting upon sleepers lying in the ground. A great many of the stables were located in sections of the city where the ground had been made by filling in swamps with street sweepings, ashes, garbage and material of that character. In stables of this description containing twenty-five horses, I consider it nothing unusual to find one or two cases of osteo-porosis every year, and I could mention probably seven or eight grocers who keep but one horse each that have lost or been obliged to dispose of two, and, in some two or three instances, three horses each during the last ten years from the disease, a large number of which were young animals examined by me for soundness before they were bought. In stables on the second floor, or stables with cellars under them, or stables built on spiles, even if on low ground where a free current of air is allowed to circulate under the floors, I have never seen a case that originated or developed there. Further than this, I have seen well-developed cases showing intermittent lameness and enlargements of the bones of the face, changed from one location to another where the change seemed to arrest the progress of the disease, where the lameness subsided and the animals remained able to do their work for years after. A client of mine, a few days ago, whose word can be relied upon, told me that a certain horse which I told him to dispose of some two years ago, having osteo-porosis so well marked that he himself noticed the enlargement of the facial bones first, and had been sold to a farmer at that time, and that he saw the horse recently, and that the swelling of the bones of the head had disappeared and that the horse was doing good service for his present owner.

As to period of incubation and length of time it takes to render the animal useless if kept in the same stable and under the same conditions, I should say from three to twelve months. If the disease can be transmitted from sire or dam to offspring, I am not prepared to say, as my practice is exclusively in the city, and the early history of the cases I have seen could not be traced. I do know of two cases, however, where mares suffering from osteo-porosis had good, healthy colts last Spring, and both colts up to this time are apparently in perfect health, and both mares are dead.

According to Williams, Varnell was the first to describe this disease, in 1860, and I consider it reasonable to suppose that it did not exist to any great extent prior to that in England. I am satisfied that there was none of it in Percival's time, for he was as careful an observer and as brilliant a writer as has ever contributed to veterinary literature. In this country I believe it to be of comparatively recent origin. The very fact that Dr. Hausman had never seen a case prior to 1879 would strongly point in this direction.

As to causes, considering the cases that have come under my observation, and more particularly the conditions and location of the stables in which these cases were found, I cannot help but conclude that a specific germ, a mineral, or perhaps, a gas, the development or generation of which is favored by certain soils and certain conditions, are the most probable causes, and that this substance, whatever it may be, finds its way into the body of animals, acts specifically upon the osseous system, and causes degeneration of a destructive character. From our present knowledge it would be folly to attempt treatment. Prevention, however, should be aimed at, and during the last six months I have made an effort to have all animals showing the slightest suspicious symptoms of osteo-porosis, removed to other stables, and in all stables where the disease appeared repeatedly, I have had the floors taken up, two or three feet of urine and manure-saturated soil removed, the old flooring boards and sleepers carefully cleaned and disinfected and a new supply of good, fresh, sandy soil put down before the floors were replaced. In cases where it was possible to have the floors raised twelve or eighteen inches from the ground, I have strongly advocated to owners the advisability of doing so. As these preventive experiments have been tried by me for the limited period of six months only, I am not prepared to say at this time that they are of any great benefit.

